# Regulation of Gonadotropin-Releasing Hormone-(1–5) Signaling Genes by Estradiol Is Age Dependent

**DOI:** 10.3389/fendo.2017.00282

**Published:** 2017-10-27

**Authors:** Bradly M. Bauman, Weiling Yin, Andrea C. Gore, T. John Wu

**Affiliations:** ^1^Department of Obstetrics and Gynecology, Uniformed Services University of the Health Sciences, Bethesda, MD, United States; ^2^Division of Pharmacology and Toxicology, Department of Psychology, Institute for Neuroscience, The University of Texas at Austin, Austin, TX, United States

**Keywords:** gonadotropin-releasing hormone, gonadotropin-releasing hormone (1–5), GPR101, GPR173, EP24.15, aging, estradiol

## Abstract

Gonadotropin-releasing hormone (GnRH) is a key regulatory molecule of the hypothalamus–pituitary (PIT)–gonadal (HPG) axis that ultimately leads to the downstream release of estradiol (E_2_) and progesterone (P). These gonadal steroids feed back to the hypothalamus and PIT to regulate reproductive function and behavior. While GnRH is thought to be the master regulator of reproduction, its metabolic product GnRH-(1–5) is also biologically active. Thimet oligopeptidase 1 (also known as EP24.15) cleaves GnRH to form GnRH-(1–5). GnRH-(1–5) is involved in regulation of the HPG axis, exerting its actions through a pair of orphan G protein-coupled receptors, GPR101 and GPR173. The physiological importance of GnRH-(1–5) signaling has been studied in several contexts, but its potential role during reproductive senescence is poorly understood. We used an ovariectomized (OVX) rat model of reproductive senescence to assess whether and how GnRH-(1–5) signaling genes in hypothalamic subnuclei change in response to aging and/or different estradiol replacement regimens designed to model clinical hormone replacement in women. We found that *Gpr101* and *Gpr173* mRNA expression was increased with age in the arcuate nucleus, while expression of *Gpr173* and *EP24.15* increased with age in the medial preoptic area. Treatment with E_2_ in younger OVX animals increased expression of *Gpr101, Gpr173*, and *EP24.15*. However, older animals treated with E_2_ showed decreased expression of these GnRH-(1–5) signaling genes, displaying an age-related decline in responsiveness to E_2_. To our knowledge, this is the first study to systematically assess the effects of age and different clinically relevant regimens of E_2_ replacement on GnRH-(1–5) signaling genes.

## Introduction

Gonadotropin-releasing hormone (GnRH) is a key regulatory molecule of the hypothalamus–pituitary (PIT)–gonadal (HPG) axis. Neurons in the hypothalamus release GnRH which acts downstream on the PIT to stimulate transcription and secretion of luteinizing hormone (LH) and follicle stimulating hormone (FSH) (1). In turn, LH and FSH stimulate follicular maturation and release of the steroids estradiol (E_2_) and progesterone (P). These steroids feed back to the HPG axis to maintain homeostatic regulation of reproductive function and behavior.

While GnRH is thought to be the master regulator of reproduction, its metabolic product GnRH-(1–5) is also shown to be biologically active. GnRH-(1–5) is produced after thimet oligopeptidase 1 (also known as EP24.15) cleaves the covalent bond linking the fifth and sixth amino acids of GnRH (2, 3). GnRH-(1–5), like its parent peptide, is involved in the regulation of the HPG axis. Both GnRH gene expression (4) and secretion (5) are stimulated by GnRH-(1–5). Additionally, the facilitation of lordosis by GnRH is mediated by its metabolism to GnRH-(1–5) (6). However, GnRH-(1–5) binds to alternative receptors than its parent GnRH peptide (6, 7). The actions of GnRH-(1–5) are mediated through a pair of orphan G protein-coupled receptors, GPR101 and GPR173 (8–10). The downstream signaling actions of GnRH-(1–5) occur through traditional G-protein signaling pathways (GPR101) and non-canonical pathways in which β-arrestin 2 is rapidly recruited (GPR173) (8, 11).

It is thought that during aging, changes in the hypothalamic GnRH system, as well as PIT and ovarian processes, are key components that contribute to reproductive senescence (12). The hypothalamic changes include decreased GnRH release and neural activation, as well as diminution of the preovulatory GnRH/LH surge. The subsequent PIT and ovarian changes result in diminished E_2_ and P secretion and changes in the positive feedback system on GnRH-induced LH surge [reviewed in Ref. (13)]. Additionally, GnRH cleavage enzyme (EP24.15) immunoreactivity within the median eminence where GnRH axons terminate is sensitive to hormonal changes, as its expression decreases from the early proestrous period (high circulating E_2_ and low LH) to the late proestrous period (low circulating E_2_ and high LH) (3). It is possible that GnRH-(1–5) may have additional peripheral effects (14). However, whether and how the GnRH-(1–5) signaling pathway changes during reproductive aging within the brain is unknown.

Utilizing a reproductive aging female rat model (15, 16), we sought to determine the impact of age and hormone treatment duration and timing on genes crucial for GnRH-(1–5) signaling, particularly in the medial preoptic area (mPOA) and arcuate nucleus (ARC) of the hypothalamus. The mPOA is of interest as GnRH cell bodies are mainly found here, and it is a major site for the regulation of reproductive function (17–19). The ARC is responsible for regulating the negative feedback response to E_2_, as well as assisting in generation of the pulsatile release of GnRH (20, 21). Importantly, *Gpr101, Gpr173*, and *EP24.15* are all expressed within these regions (22–24). In addition, gene expression within motor cortex (MC) and PIT were used as comparisons to the hypothalamus. Our goal is to provide mechanistic insights into GnRH and GnRH-(1–5) signaling pathway regulation by E_2_ deficiency and treatment during reproductive aging.

## Materials and Methods

Tissue samples assayed in this study were previously generated and utilized for separate publications (15, 16). This study utilized the cDNA generated by these previous publications.

### Animals

Female Sprague-Dawley rats (Harlan, Indianapolis, IN, USA) were purchased at 3–4 months [reproductively mature (MAT); virgin] and 10–11 months old [reproductively aging (AG); retired breeder]. On arrival, rats were pair housed at random with same-age partners in a controlled room temperature (22°C) and light cycle (12-h light, 12-h dark, lights on at 7 a.m.). Food and water were available *ad libitum*. All animal experiments were conducted following protocols approved by the Institutional Animal Care and Use Committee at the University of Texas at Austin and in accordance with The Guide for the Care and Use of Experimental Animals.

**Figure 1:**
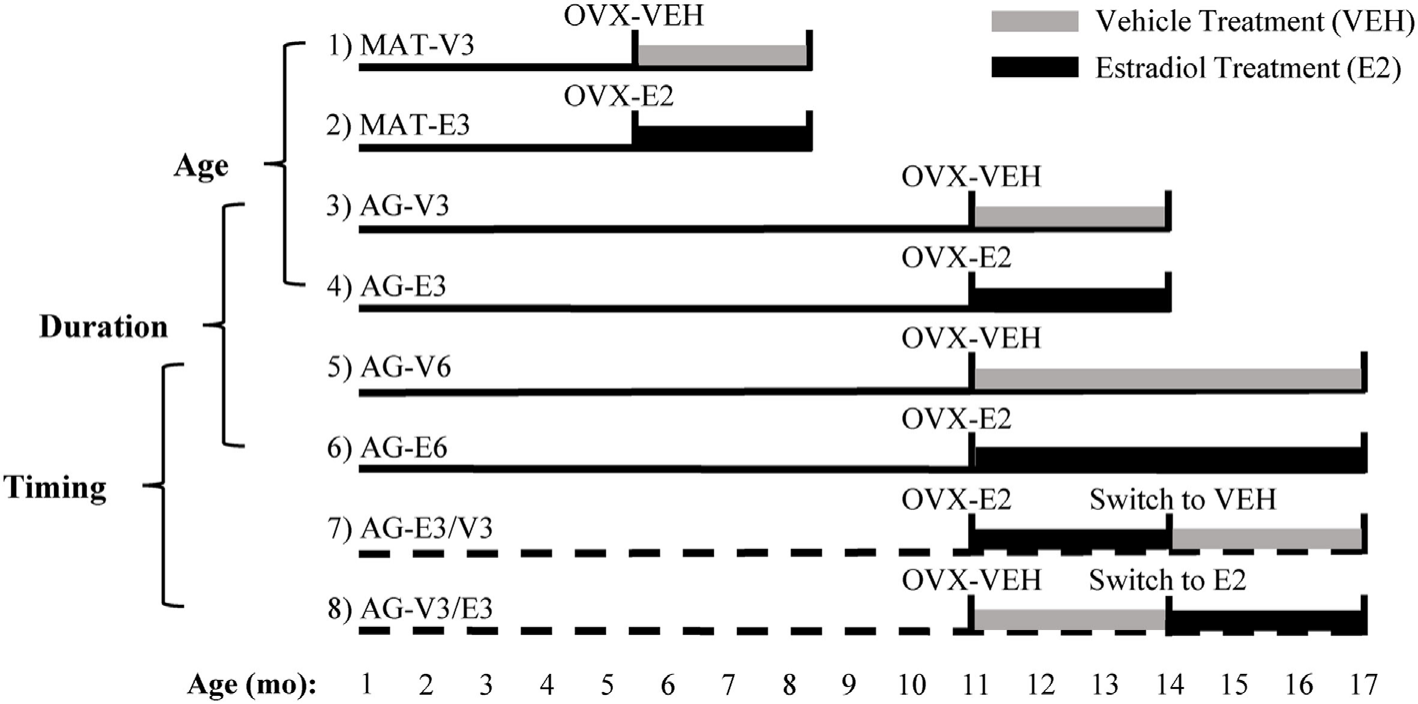
Experimental design used to study the effects of age, timing, and duration of hormone treatment. Ovariectomy (OVX) surgery, followed by vehicle (VEH) or estradiol (E_2_) capsule implantation, was performed at age 4–5 [mature (MAT)] and 11–12 months [aging (AG)]. Animals in groups 1–6 were MAT or AG rats that were given VEH or E_2_ for 3 or 6 months. Animals in groups 7 and 8 were AG rats that were given VEH or E_2_ post-OVX and then switched after 3 months to the opposite treatment for an additional 3 months. Comparisons between groups 1–4, 3–6, and 5–8 were made as described in Section “Materials and Methods.” Figure adapted from Ref. (16).

The animal procedures were previously described (15, 16). Briefly, animals were acclimated to the new housing environment for 1 week prior to 2 weeks of estrous cycle monitoring by vaginal lavage with sterile saline. Females age 3–4 months with regular 4–5 days cycles were used for the MAT group. Females age 10–11 months with regular cycles (50%), irregular estrous cycles (30%), or persistent estrus (20%) were randomly assigned to different treatments for the AG groups (Figure 1). Upon determination of estrous cyclicity, all animals underwent bilateral ovariectomy (OVX) under isoflurane inhalation anesthesia, and each animal was administered a non-steroidal anti-inflammatory drug (Rimadyl; 5 mg/kg) at the beginning of surgery for analgesia. Animals were randomly assigned to one of eight treatment groups (Figure 1, groups 1–8), allowing for the examination of different temporal regimens of hormone treatment (16). Animals within the same cage always received the same treatment.

At the time of surgery, Silastic capsules containing either 100% cholesterol (VEH) or 5% 17β-estradiol/95% cholesterol (E_2_) were implanted subcutaneously between the shoulder blades. Delivery of E_2_ *via* Silastic capsule was previously shown to last for at least 6 months without loss of integrity (25). After OVX, all animals received new identifiers to allow for blinded data collection. Groups 1 and 2 consisted of MAT animals treated with VEH or E_2_ for 3 months, respectively (MAT-V3 and MAT-E3). Groups 3 and 4 consisted of AG animals treated with VEH or E_2_ for 3 months, respectively (AG-V3 and AG-E3). Groups 5 and 6 consisted of AG animals treated with VEH or E_2_ for 6 months, respectively (AG-V6 and AG-E6). Groups 7 and 8 consisted of AG animals treated with E_2_ followed by VEH for 3 months each (group 7, AG-E3/V3) or VEH followed by E_2_ for 3 months each (group 8, AG-V3/E3).

### Tissue Collection

After 3 or 6 months of hormone treatment, animals were euthanized by rapid decapitation between 1 and 3 p.m. (4–6 h before lights off at 7 p.m.). Brains were quickly extracted and briefly cooled on ice. Coronal brain sections (eight total) were taken at 1 mm intervals throughout the entire hypothalamus using an ice-cold brain matrix (Ted Pella, Inc., Redding, CA, USA). Sections were quickly immersed in 1.5 mL of RNAlater (cat. no. AM7021M, Invitrogen, Waltham, MA, USA) and stored overnight at 4°C. After overnight storage, each section was mounted on plain glass slides and stored at −20°C before micropunching. Additionally, at time of euthanasia, the PIT was removed, immersed in RNAlater overnight at 4°C, and stored at −20°C prior to RNA extraction.

### RNA Extraction and Real-time PCR

Brains treated with RNAlater were thawed once for micropunching. Hypothalamic regions containing the mPOA (bregma −0.26 to −1.80) and ARC (bregma −2.12 to −4.52) (26) were micropunched using a 1.2-mm diameter punch (cat. no. 57399, Stoelting, Wood Dale, IL, USA). As a control (non-hypothalamic) region, MC was punched, using a 1.2-mm diameter punch, from regions related to motor control of the forelimbs and forepaws. The entire PIT was used for extraction of RNA. Total RNA, from all regions and the PIT, was extracted using the RNeasy Mini Kit (cat. no. 74104, Qiagen, Valencia, CA, USA) according to the manufacturer’s protocol. DNase digestion was performed on-column using the RNase-Free DNase Set (cat. no. 79254, Qiagen). RNA was eluted in 30 µL RNase-free water, and RNA quality and concentration were determined with the Agilent RNA 6000 Nano kit (cat. no. 5067-1511, Agilent Technologies, Santa Clara, CA, USA) on the bioanalyzer. For each region, 200 ng of total RNA was reverse-transcribed to single-stranded cDNA using a high capacity cDNA reverse transcription kit (cat. no. 4374966, Applied Biosystems, Foster City, CA, USA).

The mRNA expression of *Gpr101, Gpr173, EP24.15*, and the control gene *Gapdh* was assessed in each region by real-time PCR using the iQ SYBR Green Supermix (cat. no. 1708884, Bio-Rad, Hercules, CA, USA). Additionally, mRNA expression of *Gnrh1* and *Gnrhr* were assessed in the mPOA and PIT, respectively. Each sample was assayed in duplicate using 400 nM of the appropriate primer pair on the CFX Connect Real-time System (Bio-Rad). Mature adult male rat hypothalamus was included in each assay as an intra- (0.281 ± 0.044%) and interassay (0.601 ± 0.084%) control. The following cycling parameters were used: initial denaturation and enzyme activation at 95°C for 3 min followed by 40 cycles of denaturation (95°C, 15 s), annealing (60°C, 30 s), extension (72°C, 30 s), and reading. Melt curve analysis was conducted after each real-time reaction to demonstrate the presence of a single amplicon. Amplified products were purified using the QIAquick PCR Purification Kit (cat. no. 28104, Qiagen) and verified post-purification by agarose gel analysis and sequenced with the ABI 3500xL Genetic Analyzer (Applied Biosystems). Sequences were verified using NCBI BLAST and comparing sequences to the Reference RNA sequences (refseq_rna) database. Primers specific for *Gapdh, Gnrh1, Gnrhr, Gpr101, Gpr173*, and *EP24.15* are shown in Table 1. Relative expression of each gene was determined using the delta delta *C*_t_ (ΔΔ*C*_t_) method (27–29), normalizing each sample to *Gapdh*. In a previous publication (15), additional brain regions from these animals were analyzed and *Gapdh* was used as the housekeeping gene. In both that study and the current study, there were no effects of age or E_2_ treatment on the expression of *Gapdh*. Therefore, *Gapdh* was determined to be a valid normalizing gene. All data were expressed relative to the mature, vehicle-treated group (MAT-V3; Figure 1). For each group, cDNA for up to seven animals was analyzed, and deviation from this number in each figure is the result of lack of expression, exhaustion of samples, or the removal of outliers after analysis *via* Grubb’s test.

**Table 1:** Primer sequences used for real-time PCR.

Gene	Accession number	Primer sequence	Amplicon size (bp)
*Gapdh*	NM_017008.4	(F) 5′-GTGCCAGCCTCGTCTCATAG-3′	122
		(R) 5′-CGTTGATGGCAACAATGTCCA-3′	
*Gnrh1*	NM_012767.2	(F) 5′-GGCTTTCACATCCAAACAGAATG-3′	181
		(R) 5′-TGATCCTCCTCCTTGCCCAT-3′	
*Gnrhr*	NM_031038.3	(F) 5′-TCAGGACCCACGCAAACTAC-3′	182
		(R) 5′-CTGGCTCTGACACCCTGTTT-3′	
*Gpr101*	NM_001108258.1	(F) 5′-ATAGCCATCCTGAGCTTCGC-3′	167
		(R) 5′-CGGTGCGCTGAATAGAAAGC-3′	
*Gpr173*	NM_022255.1	(F) 5′-CGAGTATCGTCACCGCAAGA-3′	119
		(R) 5′-CAAAGCCAGCGATCCAGTTG-3′	
*EP24.15*	NM_172075.2	(F) 5′-GTGTACCAGAGGGTCGTGTG-3′	142
		(R) 5′-TGATCTTCTCCTGTGTGTCCTG-3′	

### Statistical Analyses

Statistical analyses were conducted using GraphPad Prism 6 software (GraphPad Software, Inc., La Jolla, CA, USA). Differential tissue expression of *Gpr101, Gpr173*, and *EP24.15* was compared using a one-way ANOVA followed by a Bonferroni *post hoc* test. Based on the study design (Figure 1), three different sets of comparisons were performed: (1) The effects of age (MAT vs. AG) and hormone (VEH vs. E_2_) were analyzed by two-way ANOVA (groups 1–4; Figure 1). (2) The effects of treatment duration (3 vs. 6 months) and hormone (VEH vs. E_2_) were analyzed by two-way ANOVA (groups 3–6; Figure 1). (3) The effect of the timing of hormone treatment was analyzed by a one-way ANOVA with a Bonferroni *post hoc* test (groups 5–8; Figure 1). Interactions among variables were also analyzed for the two-way ANOVA analyses. For each analysis, significant main or interaction effects were followed by a Fisher least significant difference *post hoc* test. A value of *p* < 0.05 was considered significant. *Gnrh1* expression data in the mPOA were transformed utilizing the ratio transform in GraphPad. Prior to statistical analysis, these transformed data were log-transformed for analysis. Assistance with statistical analyses was provided by the USUHS Biostatistics Consulting Center.

## Results

### Relative Abundance of GnRH-(1–5) Signaling Genes

**Figure 2:**
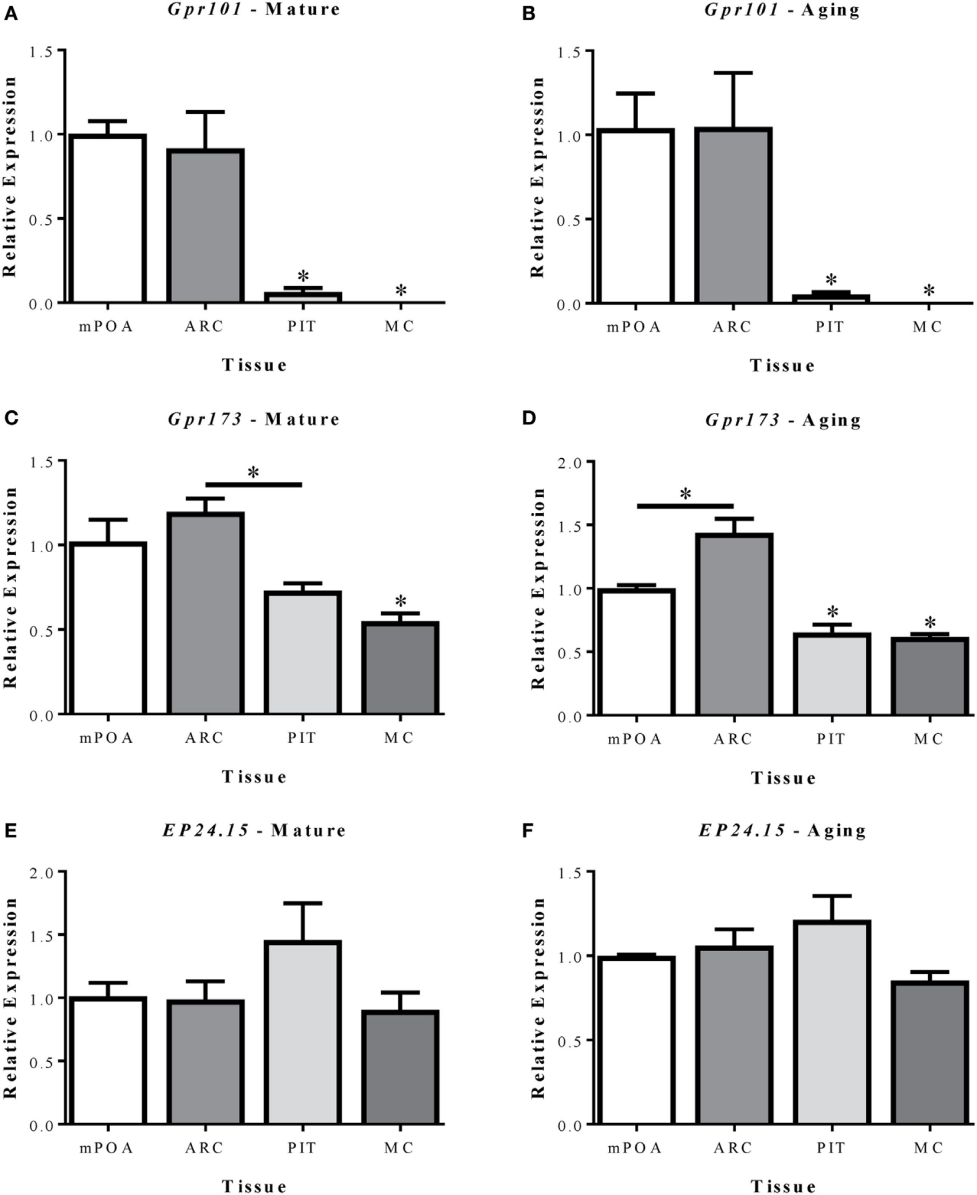
Relative mRNA expression of *Gpr101* **(A,B)**, *Gpr173* **(C,D)**, and *EP24.15* **(E,F)** in three brain regions and the pituitary of mature (7–8 months; left column) and aging (14–17 months; right column) female Sprague-Dawley rats. **(A–F)** The tissues analyzed included the medial preoptic area (mPOA), arcuate nucleus (ARC), pituitary (PIT), and motor cortex (MC). **(A,B)** Expression of *Gpr101* in mature and aging female Sprague-Dawley rats. **(C,D)** Expression of *Gpr173* in mature and aging female Sprague-Dawley rats. **(E,F)** Expression of *EP24.15* in mature and aging female Sprague-Dawley rats. Data shown are mean ± SEM (*n* = 3). *p < 0.05 versus mPOA and ARC unless otherwise specified.

The relative abundance of *Gpr101, Gpr173*, and *EP24.15* within the mPOA, ARC, PIT, and MC was first compared in mature (MAT-V3) and aging (AG-V3) rats (Figure 2). Expression of each gene is shown relative to levels in the mPOA. In both mature and aging animals, *Gpr101* expression was significantly greater in the mPOA and ARC versus the PIT and MC (Figures 2A,B, *p* < 0.05). In mature animals, the expression of *Gpr173* in the ARC was greater than in the PIT and MC (*p* < 0.05). However, in mature animals, the expression of *Gpr173* in the mPOA was only significantly increased compared with the MC (Figure 2C, *p* < 0.05). Among aging animals, the expression of *Gpr173* was greater in the ARC than in the mPOA (*p* < 0.05), and both regions had higher expression than the PIT and MC (Figure 2D, *p* < 0.05). There were no differences in *EP24.15* expression levels among tissues analyzed (Figures 2E,F). The average *C*_t_ values from the MAT-V3 group were graphed to compare the relative expression of each gene within the different tissues analyzed (Figures S1A–D in Supplementary Material).

### Effects of Age and Estradiol on GnRH-(1–5) Receptors mRNA Expression

**Figure 3:**
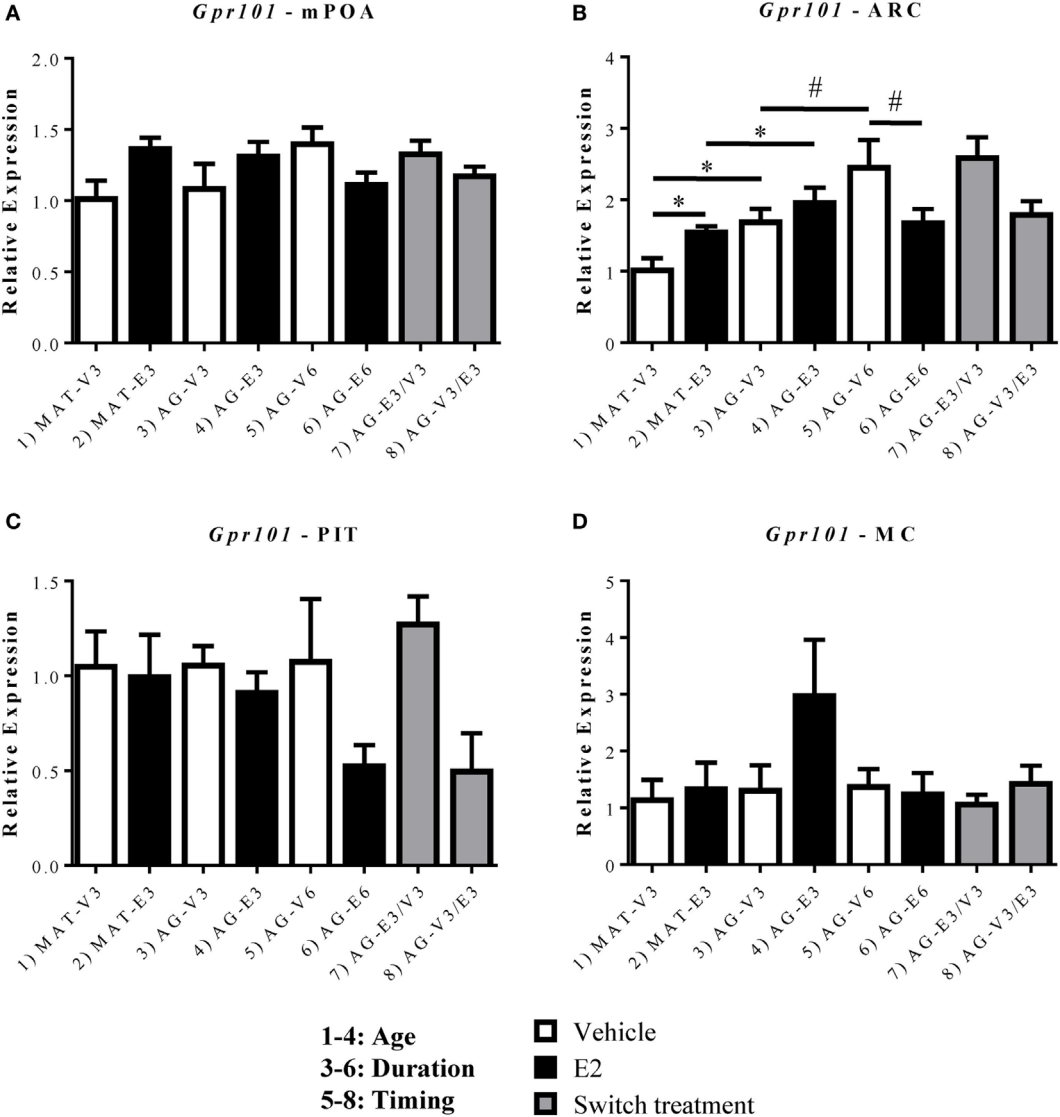
Effects of age and estradiol on mRNA expression of *Gpr101*. **(A–D)** The expression of *Gpr101* mRNA was analyzed within the **(A)** medial preoptic area (mPOA), **(B)** arcuate nucleus (ARC), **(C)** pituitary (PIT), and **(D)** motor cortex (MC). Comparisons of age were made between groups 1–4 (analysis 1), duration of hormone treatment between groups 3–6 (analysis 2), and timing of hormone treatment between groups 5–8 (analysis 3). All comparisons were made as described in Section “Materials and Methods.” Data shown are mean ± SEM (*n* = 4–7). *p < 0.05 (analysis 1); #*p* < 0.05 (analysis 2).

**Figure 4:**
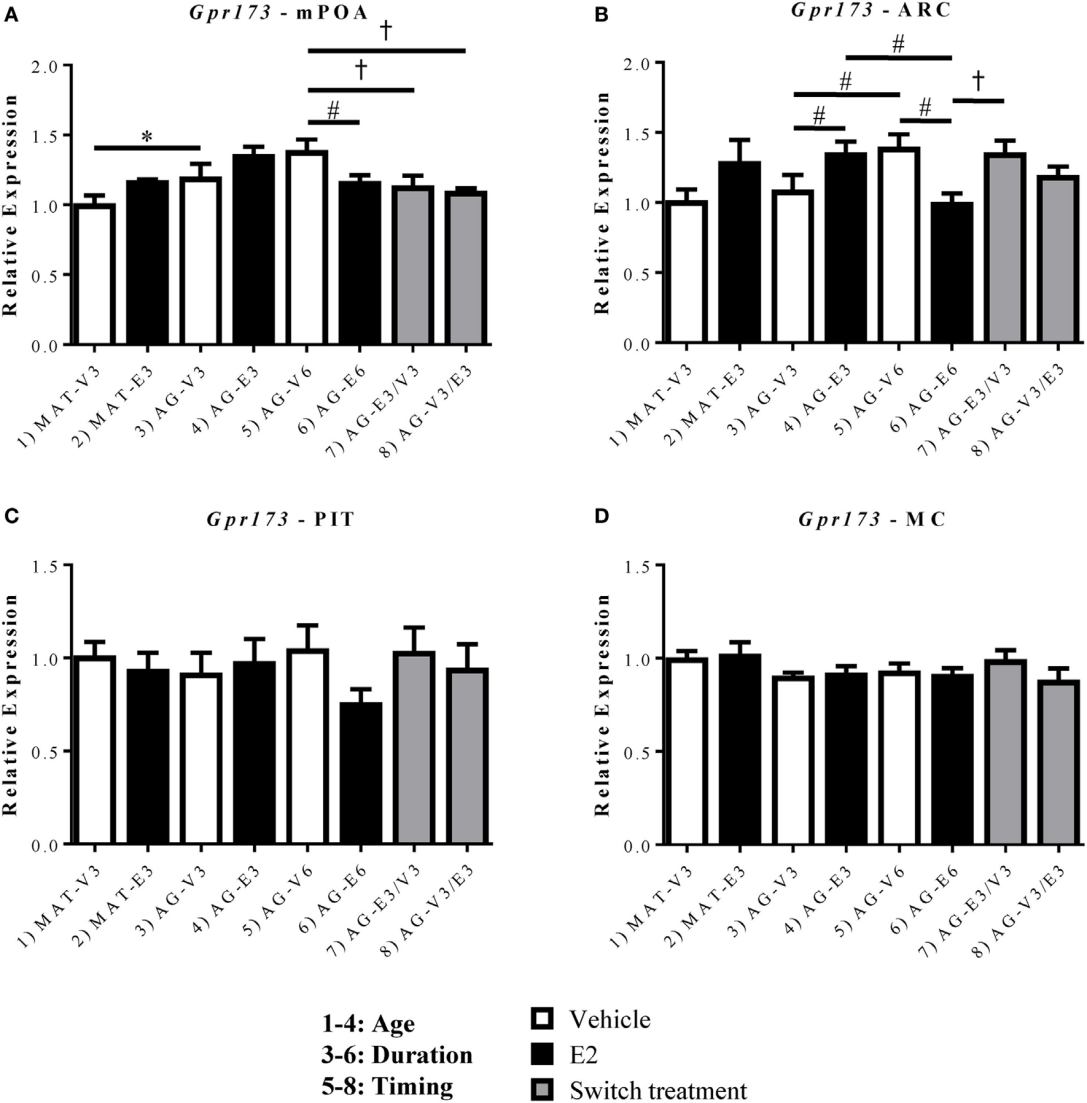
Effects of age and estradiol on mRNA expression of *Gpr173*. **(A–D)** The expression of *Gpr173* mRNA was analyzed within the **(A)** medial preoptic area (mPOA), **(B)** arcuate nucleus (ARC), **(C)** pituitary (PIT), and **(D)** motor cortex (MC). Comparisons of age were made between groups 1–4 (analysis 1), duration of hormone treatment between groups 3–6 (analysis 2), and timing of hormone treatment between groups 5–8 (Analysis 3). All comparisons were made as described in Section “Materials and Methods.” Data shown are mean ± SEM (*n* = 5–7). *p < 0.05 (analysis 1); #*p* < 0.05 (analysis 2); †*p* < 0.05 (analysis 3).

To determine the mechanisms by which GnRH-(1–5) signaling was altered after aging, ovarian hormone loss, and E_2_ treatment, we quantified mRNA expression of its receptors, *Gpr101* and *Gpr173*, within the mPOA, ARC, and PIT (Figures 3 and 4). We found no significant changes in *Gpr101* mRNA expression within the mPOA (Figure 3A). However, within the ARC, there were significant effects of age [*F*(1,22) = 15.24, *p* < 0.05], hormone [*F*(1,22) = 8.325, *p* < 0.05], and a significant interaction between treatment duration and hormone [*F*(1,23) = 5.179, *p* < 0.05]. There was a significant increase in *Gpr101* expression after 3 months of E_2_ treatment in the MAT-E3 rats (Figure 3B, *p* < 0.05). Expression of *Gpr101* consistently increased with age, as AG-V3 rats showed greater expression than MAT-V3 rats, and AG-V6 rats had greater expression than AG-V3 rats (Figure 3B, *p* < 0.05). Additionally, AG-E3 rats showed greater *Gpr101* mRNA expression than MAT-E3 rats, demonstrating the age-related increases in expression (Figure 3B, *p* < 0.05). Interestingly, treatment with E_2_ for 6 months in the aged rats (AG-E6) decreased the expression of *Gpr101* within the ARC, relative to the AG-V6 group (Figure 3B, *p* < 0.05). There were no significant changes in *Gpr101* expression within the PIT (Figure 3C), and the MC control also showed no significant changes (Figure 3D; Figure S2 in Supplementary Material).

Unlike *Gpr101, Gpr173* displayed significant effects of age and/or hormone replacement within both the mPOA and ARC (Figures 4A,B). Within the mPOA, there was a significant effect of age [*F*(1,22) = 8.485, *p* < 0.05] and a significant interaction between treatment duration and hormone [*F*(1,24) = 8.12, *p* < 0.05]. The mRNA expression of *Gpr173* increased with age between the AG-V3 and MAT-V3 rats (Figure 4A, *p* < 0.05). There were no significant effects of E_2_ in younger rats, however, those treated with E_2_ for 6 months (AG-E6) displayed decreased *Gpr173* expression relative to the AG-V6 rats (Figure 4A, *p* < 0.05). Additionally, there was no effect of the order of E_2_ treatment in the aged rats, as both the AG-E3/V3 and AG-V3/E3 rats displayed decreased *Gpr173* expression relative to the AG-V6 rats (Figure 4A, *p* < 0.05). Within the ARC, *Gpr173* expression displayed a significant interaction between treatment duration and hormone [*F*(1,24) = 15.57, *p* < 0.05; Figure 4B]. Rats treated with VEH for 6 months (AG-V6) had greater *Gpr173* expression than the AG-V3 rats (Figure 4B, *p* < 0.05). Additionally, the aged rats treated with E_2_ for 3 months (AG-E3) showed increased *Gpr173* expression compared to the AG-V3 group (Figure 4B, *p* < 0.05). As seen in the mPOA, rats treated with E_2_ for 6 months (AG-E6) actually showed a decrease in *Gpr173* relative to the AG-V6 group (Figure 4B, *p* < 0.05). Interestingly, compared to the mPOA, the timing and duration of E_2_ treatment appears to be important in the ARC, as the AG-E3/V3 rats had higher *Gpr173* expression than the AG-E6 rats, while there was no difference from the expression in the AG-V6 group (Figure 4B, *p* < 0.05). There were no significant changes in *Gpr173* expression within the PIT (Figure 4C), and the MC control also showed no significant changes (Figure 4D).

### Effects of Age and Estradiol on GnRH-(1–5) Converting Enzyme, EP24.15, mRNA Expression

**Figure 5:**
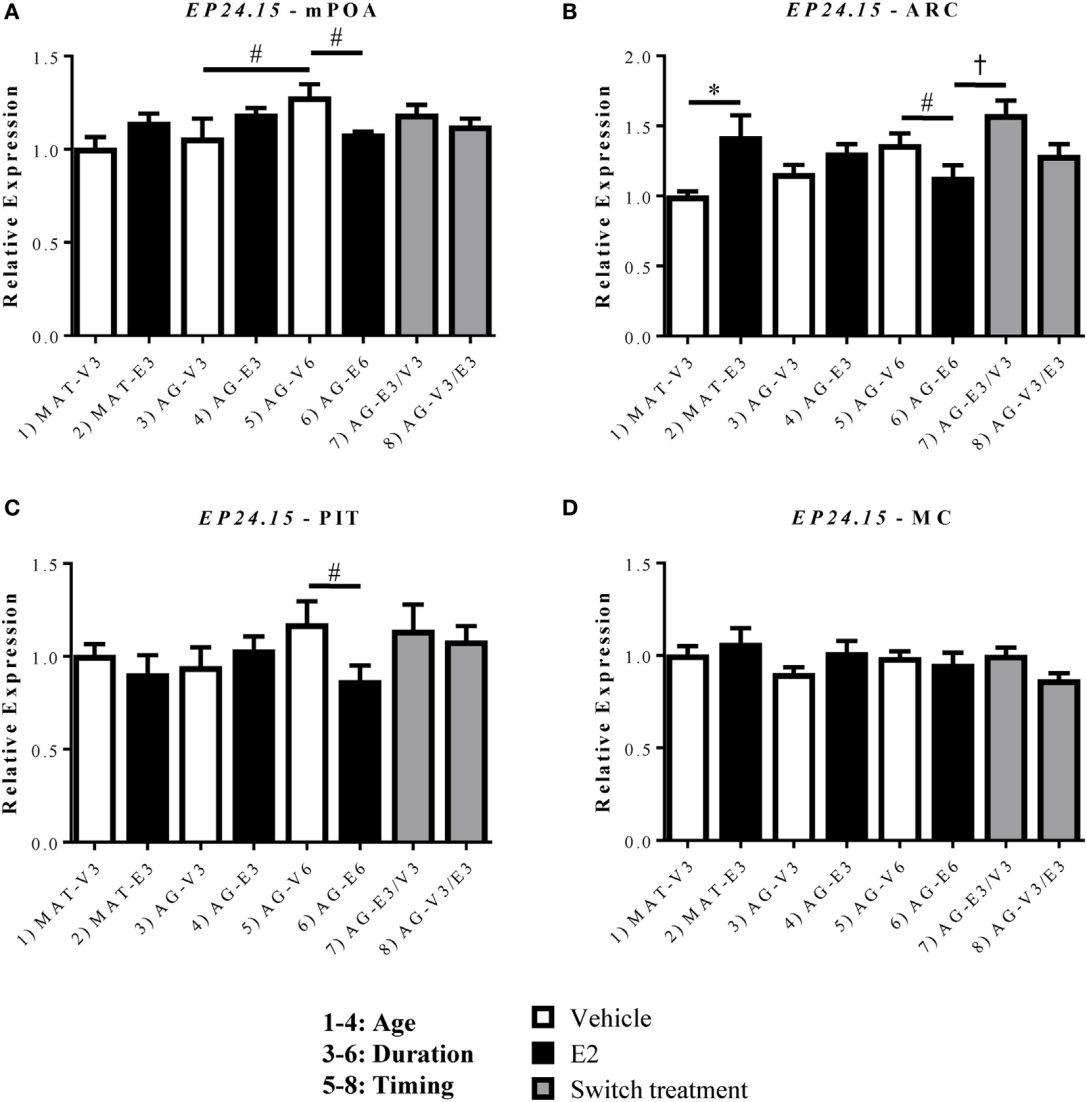
Effects of age and estradiol on mRNA expression of *EP24.15*. **(A–D)** The expression of *EP24.15* mRNA was analyzed within the **(A)** medial preoptic area (mPOA), **(B)** arcuate nucleus (ARC), **(C)** pituitary (PIT), and **(D)** motor cortex (MC). Comparisons of age were made between groups 1–4 (analysis 1), duration of hormone treatment between groups 3–6 (analysis 2), and timing of hormone treatment between groups 5–8 (analysis 3). All comparisons were made as described in Section “Materials and Methods.” Data shown are mean ± SEM (*n* = 5–7). *p < 0.05 (analysis 1); #*p* < 0.05 (analysis 2); †*p* < 0.05 (analysis 3).

*EP24.15* expression was assessed within the mPOA, ARC, and PIT (Figure 5). Within the mPOA, there was a significant interaction between treatment duration and hormone [*F*(1,24) = 6.867, *p* < 0.05]. *EP24.15* expression increased with age in the AG-V6 versus AG-V3 rats and decreased with E_2_ treatment in the AG-E6 versus AG-V6 rats (Figure 5A, *p* < 0.05). Within the ARC, there was a significant effect of hormone [*F*(1,24) = 10.51, *p* < 0.05], as E_2_ replacement increased *EP24.15* expression in the MAT-E3 versus MAT-V3 rats (Figure 5B, *p* < 0.05). As seen in the mPOA, there was a significant interaction between treatment duration and hormone [*F*(1,24) = 6.998, *p* < 0.05], and treatment with E_2_ for 6 months decreased *EP24.15* expression in AG-E6 versus AG-V6 rats (Figure 5B, *p* < 0.05). The timing of E_2_ treatment was also found to be important, as the AG-E3/V3 rats had higher *EP24.15* expression than the AG-E6 rats, yet there was no difference from the AG-V6 rats (Figure 5B, *p* < 0.05). Unlike *Gpr101* and *Gpr173, EP24.15* expression in the PIT showed a significant interaction between treatment duration and hormone [*F*(1,20) = 4.463, *p* < 0.05]. Treatment with E_2_ for 6 months decreased *EP24.15* expression in the AG-E6 versus the AG-V6 rats (Figure 5C, *p* < 0.05). There were no significant changes seen in the MC control (Figure 5D).

### Effects of Age and Estradiol on *Gnrh1* and *Gnrhr* Expression

**Figure 6:**
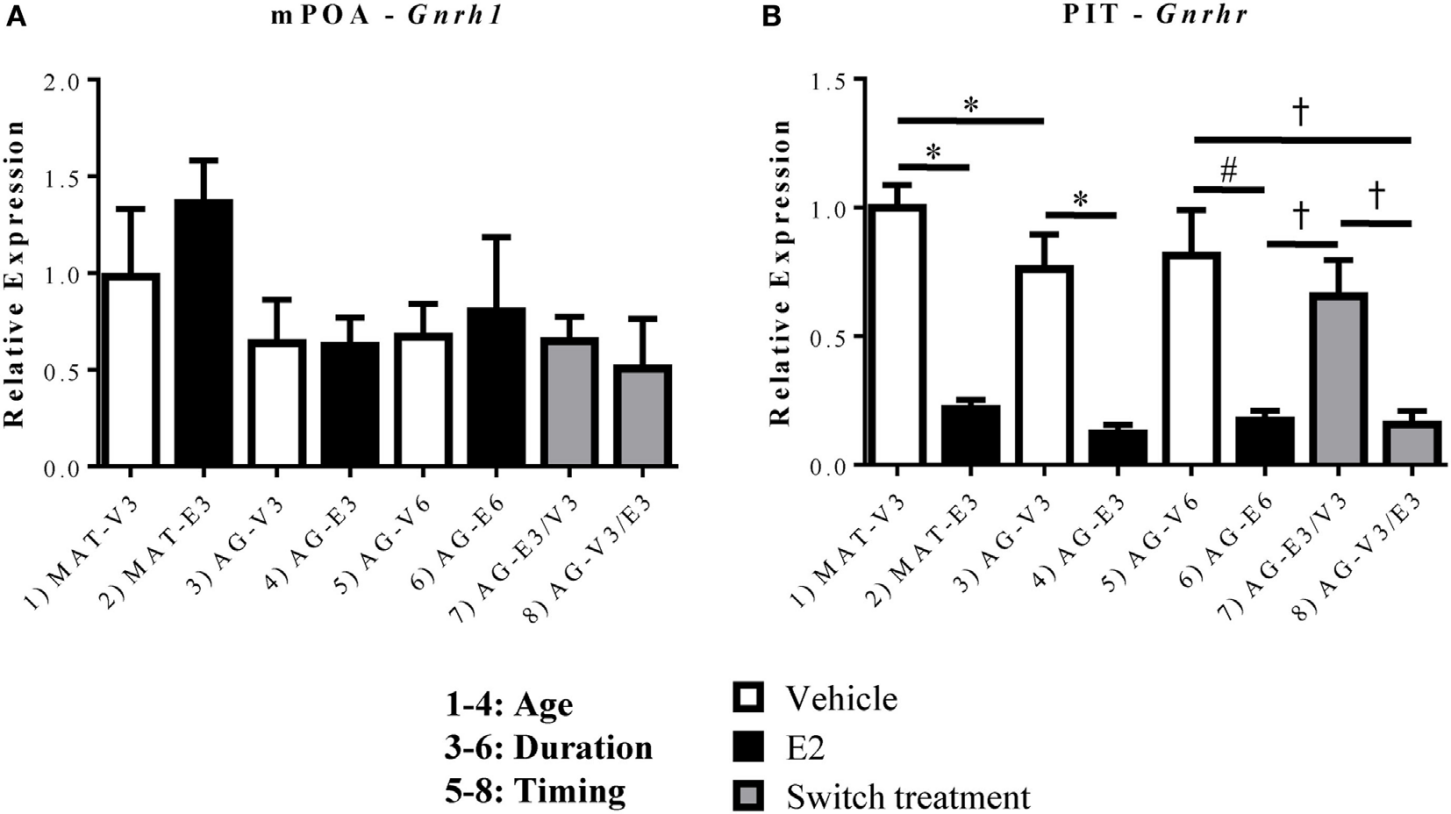
Effects of age and estradiol on mRNA expression of *Gnrh1* and *Gnrhr*. **(A)** The expression of *Gnrh1* mRNA was analyzed within the medial preoptic area (mPOA). **(B)** The expression of *Gnrhr* mRNA was analyzed within the pituitary (PIT). Comparisons of age were made between groups 1–4 (analysis 1), duration of hormone treatment between groups 3–6 (analysis 2), and timing of hormone treatment between groups 5–8 (analysis 3). All comparisons were made as described in Section “Materials and Methods.” Data shown are mean ± SEM (*n* = 6–7). *p < 0.05 (analysis 1); #*p* < 0.05 (analysis 2); †*p* < 0.05 (analysis 3).

In order to better understand the changes in key GnRH-(1–5) signaling genes, it was important to also assess whether there were changes in the expression of *Gnrh1* and its receptor, *Gnrhr*, within this paradigm. Due to these molecules’ more limited expression, this study focused on the mPOA for *Gnrh1* (where GnRH cell bodies are found) and the PIT for *Gnrhr* (where GnRH exerts its effects on the HPG axis). Within the mPOA, there were no significant changes in the expression of *Gnrh1* mRNA with age or E_2_ treatment (Figure 6A). However, there were significant effects of age [*F*(1,19) = 4.978, *p* < 0.05], hormone [*F*(1,19) = 89.43, *p* < 0.05], and treatment duration [*F*(1,20) = 39.47, *p* < 0.05] on the expression of *Gnrhr* mRNA. *Gnrhr* was consistently downregulated by E_2_ treatment at all ages and durations (Figure 6B, *p* < 0.05). As the rats aged, *Gnrhr* expression also decreased, as the AG-V3 rats showed lower expression than the MAT-V3 rats (Figure 6B, *p* < 0.05). The timing and duration of E_2_ replacement became important in the aging rats as treatment for 3 months followed by VEH for 3 months (AG-E3/V3) did not show any difference from 6 months of VEH treatment (AG-V6); however, *Gnrhr* expression in this group was significantly higher than in rats treated with E_2_ for 6 months (AG-E6) and those treated with VEH followed by E_2_ (AG-V3/E3; Figure 6B, *p* < 0.05).

## Discussion

This study analyzed the effects of aging and the timing and duration of E_2_ treatment on the expression of key GnRH-(1–5) signaling genes in the OVX rat. Gene expression of receptors that GnRH-(1–5) binds to, *Gpr101* and *Gpr173*, was affected by both age and E_2_ in the mPOA (*Gpr173*) and ARC (*Gpr101 and Gpr173*). Additionally, gene expression of the enzyme that generates GnRH-(1–5), *EP24.15*, was affected by both age and E_2_ in the mPOA and by E_2_ alone in the ARC and PIT. To our knowledge, this is the first study to systematically assess the effects of age and different clinically relevant regimens of E_2_ replacement on GnRH-(1–5) signaling genes.

### Aging Affects Relative Distribution of GnRH-(1–5) Signaling Genes

The present study showed tissue-specific expression of *Gpr101, Gpr173*, and *EP24.15* mRNA. Presently, there is a paucity in quantitation of *Gpr101* and *Gpr173* expression in specific neural nuclei. *Gpr101* mRNA is most highly expressed in the hypothalamus, specifically in the ARC (22, 30, 31) and mPOA (22, 30), across multiple species (22, 32–35). These mRNA findings have been supported by protein studies of GPR101 in the ARC and whole hypothalamus (35). Trivellin et al. also showed much higher expression of *Gpr101* mRNA in whole mouse hypothalamus versus PIT by RT-qPCR (35), a finding consistent with our study, as our results show that the expression of *Gpr101* is highest in the mPOA and ARC versus the PIT and MC. We also found that there was no apparent effect of aging on the relative distribution of *Gpr101* between the mPOA, ARC, PIT, and MC.

Similar to *Gpr101, Gpr173* mRNA has been detected in multiple species (23, 31, 33, 34, 36), with the highest expression in the hypothalamus compared with other central and peripheral regions (34). *Gpr173* mRNA in particular is highly expressed in areas related to the control of reproductive function, including areas with dense expression of estrogen receptor-α and kisspeptin, both important regulators of reproduction (23). The present study is consistent with these studies, showing that expression of *Gpr173* is highest in the hypothalamic regions we studied, including the ARC and mPOA, compared to the MC and the PIT. Detection of GPR173 protein has proven to be difficult, as effective antibodies have yet to be produced (23). Our results do show that there is an effect of age on the relative expression of *Gpr173* mRNA between tissues. The mature animals (MAT-V3) show similar expression between the mPOA and ARC, however, the aging animals (AG-V3) have significantly higher expression in the ARC compared to the mPOA. This finding should be expanded on in future studies to determine whether there is a functional role for this shift in gene expression. EP24.15 has also been detected throughout the brain and across species (3, 24, 37–40), and consistent with our results, there are no tissue-dependent differences in expression reported. Future studies are needed to examine the connection between mRNA and protein expression in this model, to determine whether the effects of age translate to the protein level.

### Aging and E_2_ Alter Expression of GnRH-(1–5) Signaling Genes

Previous studies demonstrated that GnRH-(1–5) is biologically active and has important roles in the facilitation of lordosis (6) and regulating the amplitude of GnRH pulsatile release (5). Intracerebroventricular injection of GnRH-(1–5) into the third ventricle induced lordosis, and the effects were not blocked by EP24.15 antiserum or Antide, a potent GnRH receptor antagonist (6). Additionally, treatment of hypothalamic explants, dispersed primary cells from the hypothalamus, and GT1–7 cells using GnRH-(1–5) all increased the amplitude of GnRH pulsatile release (5). Based on these results, it was important to understand the control of GnRH-(1–5) signaling gene expression after ovarian hormone loss within the mPOA, ARC, and PIT, regions crucial for HPG axis signaling.

Unlike *Gpr101*, the expression of *Gpr173* displayed significant changes in the mPOA. Within the mPOA, *Gpr173* expression increased with age after ovarian hormone loss. However, there was no significant effect of E_2_ replacement until after 6 months of treatment, at which time E_2_ decreased the expression of *Gpr173*. *Gpr173* expression 6 months post-ovarian hormone loss was also repressed by E_2_ treatment independent of the timing of treatment, as both the AG-E3/V3 and AG-V3/E3 groups had significantly lower expression than the AG-V6 animals. Similar to *Gpr173*, the expression of *EP24.15* increased with age in the mPOA. Again, there was no significant effect of E_2_ replacement until after 6 months of treatment, at which time E_2_ decreased *EP24.15* expression. Unlike the expression of *Gpr173*, there were no effects of the duration of hormone treatment on *EP24.15*.

It is within the ARC that our study identified the most significant changes in *Gpr101* mRNA expression. Our results demonstrate that as the rat ages after ovarian hormone loss, the expression of *Gpr101* mRNA increases within the ARC, and E_2_ replacement increases expression in younger animals. The expression of *Gpr173* also increased within the ARC with age after ovarian hormone loss. A similar effect was seen in the ARC for *Gpr173* and *EP24.15*, as in both cases, treatment with E_2_ in younger animals led to an increase in mRNA expression. Importantly, after 6 months of E_2_ replacement, this effect is reversed, and E_2_ actually decreases expression of *Gpr101, Gpr173*, and *EP24.15* within the ARC. These results are also similar to recently reported data that demonstrated a reversal in E_2_-induced gene expression in castrated young and aging male rats (41). Interestingly, the expression of *Gpr173* and *EP24.15* within the ARC was found to be dependent on the duration of E_2_ treatment after long-term ovarian hormone loss. Animals treated with E_2_ then switched to VEH (AG-E3/V3) displayed a significant increase in mRNA expression versus those treated with E_2_ for 6 months (AG-E6). This is an interesting result, as it differs from the effects in the mPOA for both genes. It appears that there are some similarities between *Gpr101, Gpr173*, and *EP24.15* in the ARC, however, after prolonged ovarian hormone loss, the effects of E_2_ replacement on *Gpr173* and *EP24.15* are tissue dependent. Identifying the functional significance of this tissue-dependence will be crucial to future studies.

Serum hormone levels in the rats used in the present study were measured previously. Serum LH levels were lowered by E_2_ treatment, independent of age, consistent with expected estrogen negative feedback effects (16). Similarly, *Gnrhr* expression in the PIT was significantly decreased with E_2_ treatment, independent of age, as shown in previous studies (42, 43). By contrast, our current data on the expression of *Gnrh* mRNA in the mPOA demonstrated no significant changes in expression with E_2_ treatment or age; however, previous work (in intact female rats) has shown that *Gnrh* mRNA levels may change independently of transcription and secretion (44). Previous work in OVX animals undergoing E_2_ treatment has also shown either no change or a small, significant decrease in *Gnrh* mRNA expression between young and middle-aged OVX animals (45, 46). The finding of little change in *Gnrh* expression, in the context of significant decreases in *Gnrhr* mRNA levels with E_2_ treatment, suggests the possibility of a reduced role for GnRH and an increased role for its metabolite GnRH-(1–5), as well as the potential for altered GnRH release at the median eminence.

This study assessed the effects of age, hormone, treatment duration, and the timing of treatment on the expression of key GnRH-(1–5) signaling genes. The serum E_2_ levels for the rats studied (see Figure 1 for group numbering), as published previously (15, 16), are as follows (mean ± SEM, in pg/mL): (1) 24 ± 3, (2) 105 ± 11, (3) 20 ± 1, (4) 63 ± 7, (5) 17 ± 2, (6) 63 ± 5, (7) 20 ± 2, and (8) 64 ± 6. The potential mechanisms by which E_2_ regulates the expression of *Gpr101* and *Gpr173*, the receptors for GnRH-(1–5), remain to be discovered. Evaluation of the known promoter sequences suggests no known classical estrogen response elements (EREs). However, this does not rule out the possibility of putative weak estrogen responsive motifs, or ERE-independent signaling through association with other DNA-binding transcription factors. The evaluation of the promoters for *Gpr101* and *Gpr173* is the subject of ongoing projects. Despite this lack of apparent EREs, these genes were shown to be responsive to E_2_ within the current study. The most intriguing E_2_ effects were seen in *Gpr173* expression in the ARC. It was here that AG-E3 animals displayed an increase in expression with E_2_ treatment, while AG-E6 animals displayed a decrease (serum E_2_ levels of 63 ± 7 and 63 ± 5 pg/mL, respectively), versus their respective vehicle controls. Since the AG-V6 animals already displayed increased *Gpr173* expression compared to AG-V3 animals, these effects may be a result of aging and a gain or loss of specific feedback mechanisms. Effects like these, as well as the mechanism of E_2_-responsiveness of these receptors, are yet to be elucidated.

## Conclusion

The results reported here are the first to systematically assess the effects of E_2_ replacement regimens on the mRNA expression of *Gpr101, Gpr173*, and *EP24.15* within tissues crucial to HPG regulation. Previous work has shown that OVX followed by immediate implantation with E_2_ in young rats mimics the changes in GnRH neurons and LH surges that occur at middle age in intact rats (47, 48). As such, our results suggest that rats entering middle age have increased expression of *Gpr173* and *EP24.15* in the mPOA and increased expression of *Gpr101* and *Gpr173* in the ARC. Future studies should examine the protein expression of these genes within a similar paradigm and begin to delineate the functional significance of these changes. Recent work has demonstrated that *Gpr173* mRNA is expressed in both immortalized hypothalamic GnRH and kisspeptin (Kiss1) neurons (49), a finding that should be confirmed by protein colocalization studies *in vivo*. The immunoreactivity of EP24.15 was also recently examined in relation to GnRH and Kiss1. Woitowich et al. found that EP24.15 was colocalized with GnRH neurons in the mPOA and both the internal and external zones of the median eminence in adult male rats (50). The authors also found that EP24.15 immunoreactivity was colocalized with that of Kiss1 within the ARC in metestrous female rats, however, no regions of colocalization were seen in the anteroventral periventricular nucleus (AVPV) (50), despite the identification of Kiss1 mRNA in this region (51). The localization of GPR101 in relation to GnRH or Kiss1 neurons is still to be determined.

The importance of the colocalization of EP24.15 and GPR173 with GnRH and Kiss1 neurons is slowly emerging as recent research suggests a more complex regulation of reproduction and GnRH release by a multitude of factors. Aside from cleaving GnRH to GnRH-(1–5), EP24.15 is responsible for the cleavage of Kiss1 (50) and Phoenixin (PNX) (52), both of which are peptides capable of stimulating LH secretion *in vivo* (23, 53). Immunohistochemical analysis has found PNX expressed in multiple regions of the hypothalamus, including the ARC and AVPV, both of which contain Kiss1 neurons (54). Further, these regions are particularly important for Kiss1 signaling in the female rat, as Kiss1 neurons in both the ARC and AVPV project to and modulate the negative and positive feedback effects of estrogen on GnRH neurons, respectively. Understanding the interplay between Kiss1, PNX, and GnRH-(1–5) within intact models of aging will be crucial to determining the role of each in the regulation of GnRH expression and secretion, and ultimately, their combined changes that lead to reproductive senescence.

In summary, we have systematically assessed the change in expression of genes crucial to GnRH-(1–5) signaling in response to aging and different estradiol replacement regimens designed to model clinical hormone replacement in women. Examining expression of GnRH-(1–5) signaling genes in the mPOA, ARC, and PIT is crucial as these regions are associated with regulation of GnRH (as well as LH and FSH) *via* the kisspeptin pathway [reviewed in Ref. (55).]. As the female rat ages, the E_2_-induced increases in GnRH-(1–5) signaling genes disappear, and E_2_ treatment eventually decreases the expression of *Gpr101, Gpr173*, and *EP24.15* in both the mPOA and ARC. Use of this rat model may be clinically relevant, as the primary outstanding question of the Women’s Health Initiative is determination of the optimal temporal E_2_ regimen to benefit health and well-being in women. Further studies are needed to determine the potential overlap between GnRH-(1–5) signaling, and other components of the reproductome, including Kiss1 and PNX.

## Ethics Statement

This study was carried out in accordance with the recommendations of The Guide for the Care and Use of Experimental Animals. The protocol was approved by the Institutional Animal Care and Use Committee at the University of Texas at Austin.

## Author Contributions

Conception or design of the study: WY, AG, and TW. Data collection: BB, WY, and AG. Data analysis and interpretation: BB, WY, AG, and TW. Drafting and critical revision of the article: BB, WY, AG, and TW. Final approval of the version to be published: WY, AG, and TW.

## Disclaimer

The opinions or assertions contained herein are the private ones of the authors and are not to be construed as official or reflecting the views of the Department of Defense or the Uniformed Services University of the Health Sciences.

## Conflict of Interest Statement

The authors declare that the research was conducted in the absence of any commercial or financial relationships that could be construed as a potential conflict of interest.
