# Supplementary material for: Regulation of Gonadotropin-Releasing Hormone-(1–5) Signaling Genes by Estradiol Is Age Dependent
Source: Front Endocrinol (Lausanne). 2017 Oct 27;8:282. doi: 10.3389/fendo.2017.00282 (PMC5663685; doi:10.3389/fendo.2017.00282)
Supplement: Supplementary file 1 [file data_sheet_1.docx]

Supplementary Material

Regulation of GnRH-(1-5) signaling genes by estradiol is age-dependent

Bradly M. Bauman, Weiling Yin, Andrea C. Gore, T. John Wu*

***Correspondence:** Dr. T. John Wu: twu@usuhs.mil

# Supplementary Figures


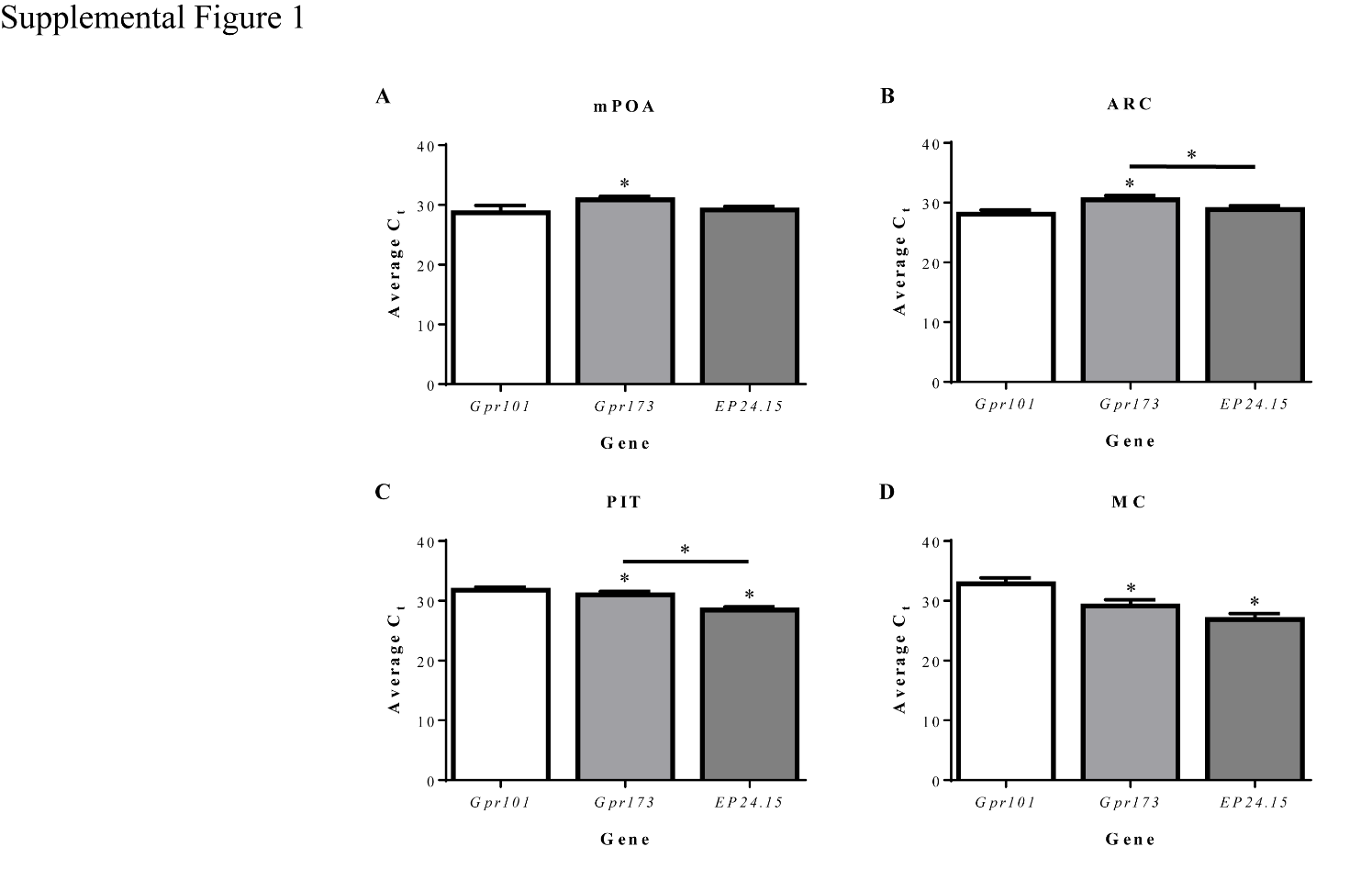


**Supplementary Figure 1.** **Average cycle threshold values (C_t_) for *Gpr101*, *Gpr173*, and *EP24.15* in 3 brain regions and the pituitary of female Sprague-Dawley rats. (A-D)** The average cycle threshold (C_t_) values of *Gpr101*, *Gpr173*, and *EP24.15* were plotted for the MAT-V3 animals. The relative expression, based on C_t_ values, was compared between the 3 genes within the **(A)** medial preoptic area (mPOA), **(B)** arcuate nucleus (ARC), **(C)** pituitary (PIT), and **(D)** motor cortex (MC). Data shown are mean ± SEM (n = 6-7). Average C_t_ values were compared via one-way ANOVA with a Bonferroni *post hoc* test. **p* < 0.05 versus *Gpr101* unless otherwise specified.


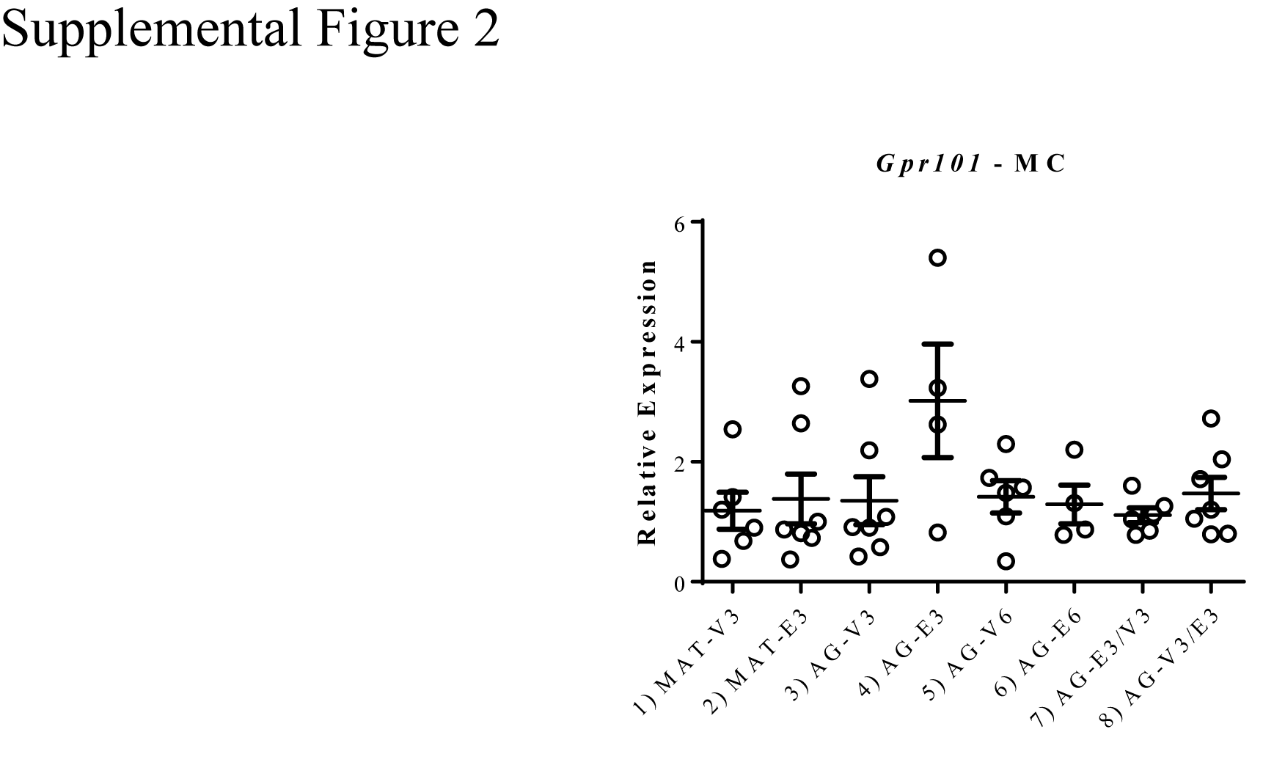


**Supplementary Figure 2. Box plot to clarify the expression of *Gpr101* within the motor cortex.** The expression of *Gpr101* mRNA was analyzed with the motor cortex (MC). There were no significant differences between groups, and low sample numbers in certain groups prevented the removal of potential outliers. Data shown are mean ± SEM (n = 4-7).
